# Millennials as Caregivers: Results From the BRFSS, 44 States, DC, and Puerto Rico, 2015-2018

**DOI:** 10.1093/geroni/igaa057.1131

**Published:** 2020-12-16

**Authors:** Nia Reed, Erin Bouldin, Christopher Taylor, Lisa McGuire

**Affiliations:** 1 Centers for Disease Control and Prevention, Atlanta, Georgia, United States; 2 Appalachian State University, Boone, North Carolina, United States

## Abstract

Millennials (born 1981-1996), now outnumber Baby Boomers. Millennials have the unique task of potentially providing care for their own children, their aging parents, and grandparents, making them the first “club sandwich” generation. We utilized data from 44 states, the District of Columbia, and Puerto Rico from the 2015-2018 Behavioral Risk Factor Surveillance System to estimate the prevalence of informal caregiving among Millennials (based on age at the time of survey completion: 18-34 in 2015, 19-35 in 2016, 20-36 in 2017, and 21-37 in 2018). We conducted log-binomial regression analyses to compare mental and physical health among Millennials, adjusting for age, sex, education, and race/ethnicity. Among 37,780 Millennials included, 18.7% provided regular care/assistance to a friend/family member with a health problem, long-term illness, or disability in the past month. Of these caregivers, 36.3% provided the most care to a parent/parent-in-law and 21.8% to a grandparent. Half of Millennials had ≥1 children under age 18 in their home, regardless of caregiving status. In adjusted models, Millennial caregivers had higher prevalence of frequent mental distress [≥14 days in the past 30 days when mental health was not good] (prevalence ratio [PR]=1.72, 95% confidence interval [CI]: 1.50-2.00, p<0.001), higher prevalence of fair/poor health (PR=1.42, 95%CI: 1.20-1.68, p<0.001), and higher prevalence of ≥1 chronic health conditions [current asthma, arthritis, cancer, cardiovascular disease, diabetes, or COPD] (PR=1.69, 95%CI: 1.51-1.90, p<0.001) than non-caregivers. Nearly one in five Millennials was providing informal care, and Millennial caregivers reported significantly worse mental and physical health than their non-caregiving peers.

